# Health Protection of CT Radiographers During the Outbreak of COVID-19: Application of Automatic Positioning Technology for Relocatable CT in the Fang Cang Hospital

**DOI:** 10.3389/fmed.2021.659520

**Published:** 2021-06-04

**Authors:** Shan Jiang, Zhongbiao Jiang, Li-Hua Luo, Kun Yu, Yeyu Cai, Xingzhi Xie, Wei Zhao, Weijun Situ, Jun Liu, Zhu Chen

**Affiliations:** ^1^Department of Radiology, The Second Xiangya Hospital, Central South University, Changsha, China; ^2^National Emergency Medical Team, The Second Xiangya Hospital, Central South University, Changsha, China; ^3^Department of Operation, The Second Xiangya Hospital, Central South University, Changsha, China

**Keywords:** automatic positioning technology, COVID-19, Fang Cang hospital, health protection, relocatable CT

## Abstract

**Background:** To investigate the value of automatic positioning technology in improving the protection of radiographers in the relocatable CT room of a Fang Cang hospital during the outbreak of coronavirus disease 2019 (COVID-19).

**Methods:** The National Emergency Medical Team of our hospital assumed command of Wuchang Fang Cang Hospital and treated confirmed COVID-19 patients with mild symptoms. Relocatable CT was used to examine patients in this hospital. Automatic positioning technology was applied to avoid close contact between medical staff and patients and to protect medical staff more effectively.

**Results:** Seven hundred lung CT scans acquired from 269 patients were completed from February 17 to 26, 2020 with automatic positioning technology for relocatable CT in a Fang Cang hospital. All scans were conducted successfully using automatic positioning technology. All patients entered the scanning room from a separate door. All the position lines were accurate, and all images met the requirement for diagnosis of COVID-19, with satisfied quality. None of our medical staff had any close contact with patients.

**Conclusion:** Automatic positioning technology applied to relocatable CT can minimize the close contact between technologists and patients and effectively improve the protection of medical staff without sacrificing image quality.

## Introduction

In December 2019, the outbreak of coronavirus disease 2019 (COVID-19) was reported in Wuhan City, Hubei Province, China (1). Due to its high infectivity and lethal potential, COVID-19 quickly spread worldwide (2, 3). The number of confirmed patients rapidly increased, and not all patients could be treated in the hospital in a timely manner, which could lead to cross-infection or secondary infection. In this context, the outbreak of COVID-19 may be exacerbated in a short time. Meanwhile, medical workers treating a substantial number of patients every day are facing extreme danger of infection. As of February 15, 2020, healthcare workers account for 1,716 confirmed cases of COVID-19, including 6 deaths in China (4). To treat confirmed patients in a timely manner and better control the outbreak, the Chinese government decided to build mobile cabin hospitals as temporary treatment centers in Wuhan, named Fang Cang hospitals. These mobile cabin hospitals were transformed from large indoor venues, such as stadiums or convention centers, into temporary hospitals that met the requirements of health protection for infectious diseases. Fang Cang hospitals were used to treat confirmed mild or common types of patients with COVID-19 (5).

Although the real-time reverse-transcriptase polymerase chain reaction (RT-PCR) assay remains the gold standard for the diagnosis of COVID-19, false-negative issues, low stability, and long test times relatively limit its application in clinical practice. CT, the most frequently used modality in clinical practice, could provide important imaging information for the detection, follow-up, and prognosis of COVID-19 (6). Relocatable CT is the best substitute for routine CT to be installed in mobile cabin hospitals to date (7). The protection of medical staff in the mobile cabin hospital during relocatable CT scanning matters, which requires high standard protective measures. Automatic positioning technology used in relocatable CT plays a vital role in the protection of medical staff and substantially avoids cross-infection.

Thus, the aim of the study was to introduce our experience in the protection of medical staff operating relocatable CT in a Fang Cang hospital during the outbreak of COVID-19 and provide a reference for peers who may use this equipment in the future.

## Materials and Methods

This study was approved by Medical Ethical Committee (Approval No. 2020002), which waived the requirement for patients' informed consent referring to the CIOMS guideline.

### Study Population

The National Emergency Medical Team of our hospital assumed command of Wuchang Fang Cang Hospital and treated confirmed COVID-19 patients with mild symptoms. From February 17 to 26, 2020, 700 lung CT scans of 269 patients completed using automatic positioning technology were included in our study.

### Relocatable CT

**Figure 1:**
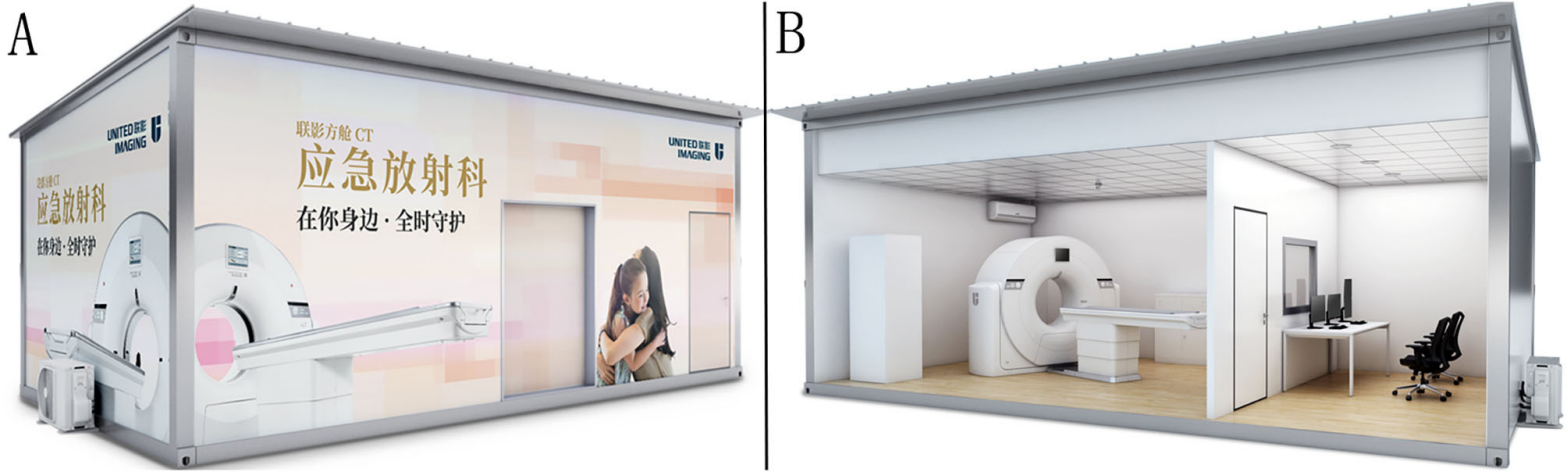
The structure of the relocatable CT system. **(A)** The outward appearance of the relocatable CT system. **(B)** The interior structure of the relocatable CT system. The figures were provided through the courtesy of Shanghai United Imaging Healthcare Co., Ltd.

A relocatable CT system is a kind of temporary large medical equipment with the same internal structure as a routine CT system (Figure 1). It contains a series of necessary facilities, including an independent scanning room, a controlling room, and an ultraviolet disinfection device. The relocatable CT can be not only installed and dismantled quickly but also transferred easily, with the advantage of an independent box-like design. The scanning room covers an area of only ~20 m^2^ and can be conveniently used with electricity. In addition, the waterproofness, thermal insulation, and constant temperature make it suitable for different extreme environments to fight the outbreak of major infectious diseases or execute rescue work.

### Health Protection

Although a relocatable CT system is not installed and used in conventional medical institutions, protective measures require the same standard during the outbreak of COVID-19 (8–10).

### Health Protection of Medical Staff

CT radiographers should apply necessary protection including disposable work caps, protective glasses or masks (anti-fog type), medical protective masks (N95), protective clothing, isolation clothing, disposable latex gloves, and disposable shoe covers (11). They must apply hand hygiene strictly according to the national hygienic standard. All protective supplies must be changed if radiographers change shifts. The health protection of medical staff must be managed strictly in accordance with the Medical Waste Management Regulations and Medical Waste Management Measures of Medical Institutions.

Radiologists review the CT images far away from the relocatable CT or review CT images online to minimize the number of medical staff entering the relocatable CT. Traditional CT requires a radiographer to position the machine in the scanning room, which inevitably leads to close contact with patients (12, 13). The mobile cabin hospital implements paperless communication to reduce unnecessary contact between medical staff and patients. An electronic communication system was applied instead.

### Prevention Measures for The Equipment and Environment

The equipment in the relocatable CT system should be wiped and disinfected with 75% ethanol at least two to four times per day. Disposable materials should be used to remove pollutants when visible pollutants are present, and then routine disinfection should be performed.

The floor of the scanning room and controlling room shall be disinfected with 2,000 mg/L chlorine disinfectant (14). When there are visible pollutants, disposable absorbent material shall be used to remove the pollutants completely, and then disinfection shall be performed at least twice per day.

A circulating air disinfector is used for continuous disinfection during operating hours, and a hydrogen peroxide air disinfector is used for spray disinfection after work is completed. An ultraviolet radiation system is used continuously for disinfection when no one is in the room four times per day (lasting for 60 min each time).

### Management of Patients

All patients should enter and exit the imaging examination area through a special channel wearing masks and try to avoid coughing during the whole process of examination.

According to the guideline from the China National Health Commission, patients with negative nucleic acid test obtained for two consecutive respiratory pathogens (sampling interval ≥1 day) need to undergo a follow-up CT to determine whether they are eligible for discharge (15). Because these patients had PCR results turned negative, they were considered possible recovered cases. Therefore, they have priority for examination and should undergo CT scans separately from those with positive RT-PCR results to avoid possible cross-infection. Also, the scanning room should be sterilized before and after each examination for every individual.

### Application of Automatic Technology

To avoid close contact between radiographers and patients as much as possible, automatic positioning technology was applied to the relocatable CT system in the Fang Cang hospital.

### Introduction of Automatic Positioning Technology

**Figure 2:**
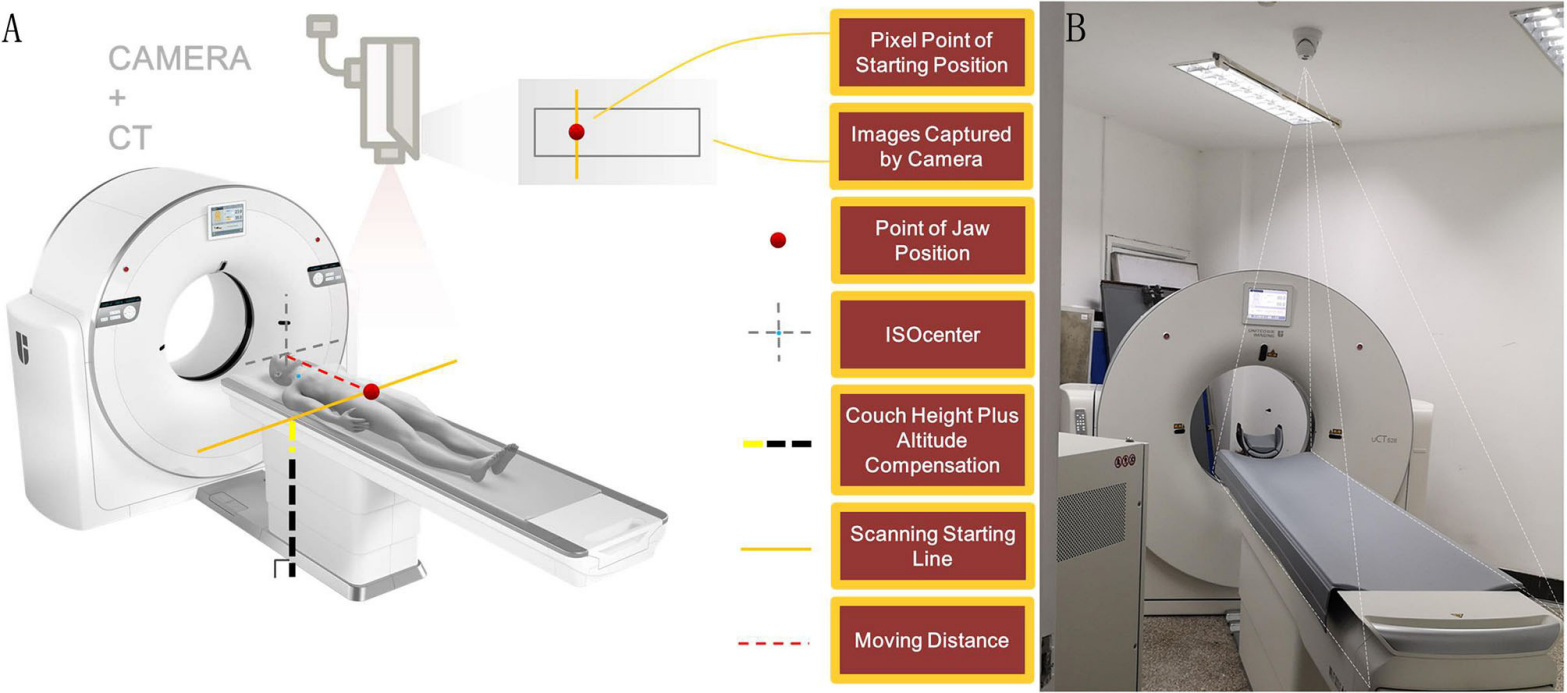
Camera-based intelligent auxiliary positioning system. **(A)** The working principle of the camera-based intelligent auxiliary positioning system. **(B)** The relocatable CT system with AI positioning technology in Wuchang Fang Cang Temporary Hospital at Hongshan Stadium in Wuchang district, Wuhan. The figures were provided through the courtesy of Shanghai United Imaging Healthcare Co., Ltd.

The automatic positioning technology system includes camera-based intelligent auxiliary positioning (Figure 2) and positioning box self-adaption.

#### Camera-Based Intelligent Auxiliary Positioning

After patients remained in the examination bed with head-first supine position, the camera can automatically detect the natural image of patients intelligently and then calculate the scanning range according to the examination site and the body type of different patients. The positioning information of the patient will be presented to the radiographer on a synchronized screen. Radiographers can adjust the position parameters or confirm them directly and then move the examination bed using a controlling button to finalize the patient's positioning.

#### Positioning Box Self-Adaption

Automatic positioning system can identify and segment the structure in the positioning image, adjust the range of the positioning box according to the scanning area, and further display the shape of the positioning box to the radiographer. Radiographers can adjust the position if needed or confirm it directly and then start the scanning.

### Operation Process

**Figure 3:**
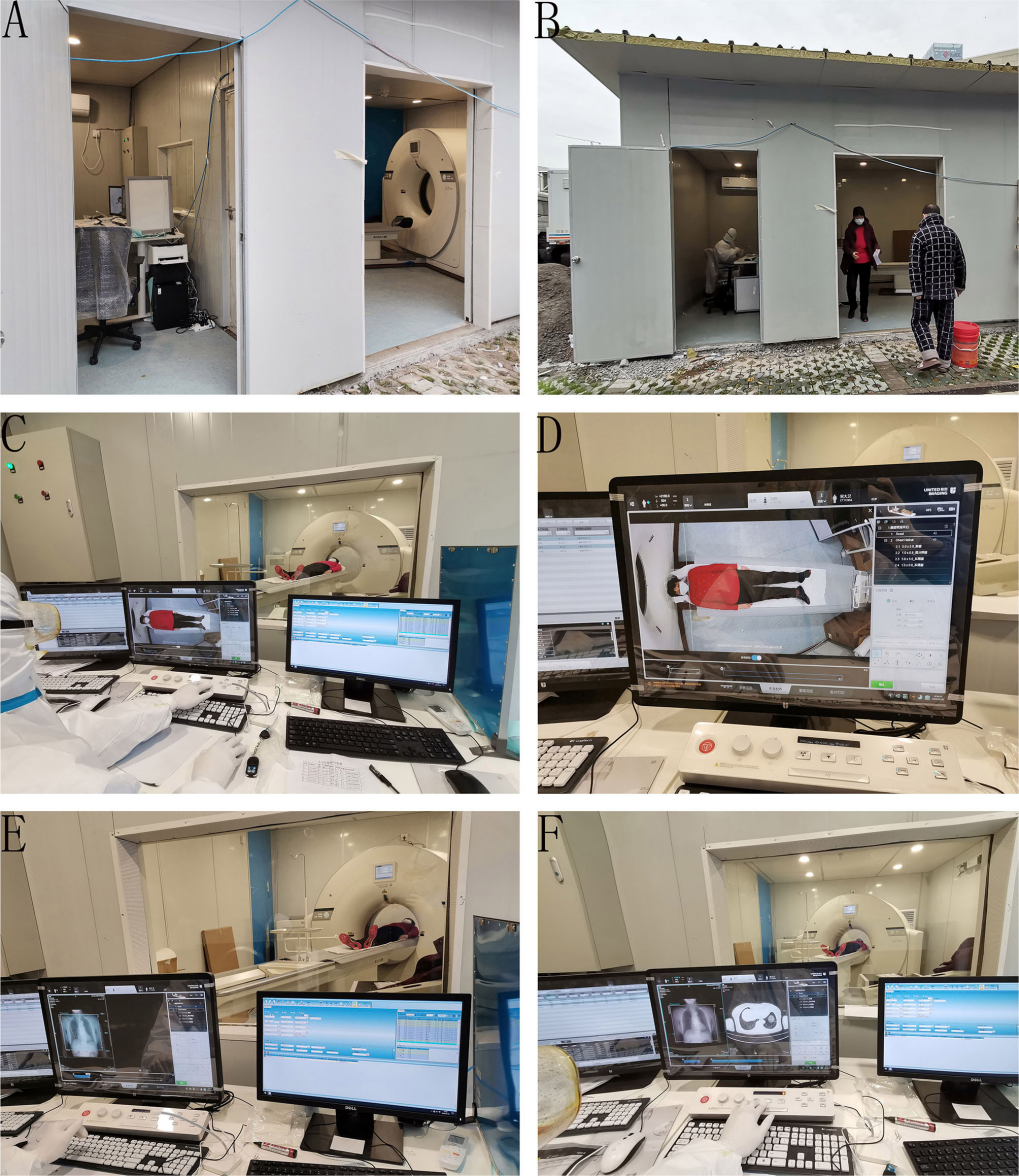
Operation process of relocatable CT aided by automatic positioning technology. **(A)** The inner structure of the relocatable CT system. **(B)** The patients enter the scanning room from a separate door by themselves. **(C)** The examination bed moves to the ideal position automatically with the help of the camera-based intelligent auxiliary positioning system. **(D)** The patient's real-time image is transmitted to a computer screen in the controlling room. **(E)** The automatic positioning system determines the scan range automatically. **(F)** The technologist starts scanning.

The patients enter the scanning room from a separate door by themselves or with the help of medical workers (Figures 3A,B). Radiographers confirmed patient's information and instructed them to lie on the examination bed through a microphone in the controlling room.

With the help of the camera-based intelligent auxiliary positioning system, the examination bed moved to the ideal position automatically according to the patient's size and the area to be examined. Then, the patient's real-time image was transmitted to a computer in the controlling room, and the technologist determined whether fine-tuning was required (Figures 3C,D).

After the radiographer confirmed the patient's position and finished the scanning of the positioning image, the automatic positioning system started the positioning box self-adaptive function and automatically set up the scan range. Radiographers could fine-tune the range or confirm it directly and then start scanning (Figures 3E,F).

After finishing the scanning, the radiographer moved the examination bed using a controlling button. The patient left the scanning room according to the radiographer's instructions.

### Scanning Parameters

All included patients were examined with the following scanner: uCT-550 (Shanghai United Imaging Healthcare Co., Ltd.). The parameters were as follows: spiral scanning, 100–120 kV or automatic tube voltage, intelligent milliampere seconds (50–350 mAs), 0.5–1.5 mm collimator width, 1–5 mm layer thickness, and layer spacing.

### Evaluation of Automatic Positioning Technology

We designated the inner positioning line aligned with the lower edge of the mandible and the horizontal positioning line parallel with the mid-axillary line as accurate position.

According to the expert consensus, we defined the accurate scanning range as from the apex pulmonis to the right costophrenic angle.

Imaging quality were evaluated by a senior radiologist. We designated images be of high quality with following points: (1) images were clear without artifacts; (2) scanning range was accurate; (3) scanning parameters were consistent with conventional pulmonary CT.

## Results

From February 17 to 26, 2020, 269 patients (166 women, 103 men) diagnosed with mild or common types of COVID-19 admitted to Wuchang Fang Cang Hospital were included in this study. The demographic characteristics are shown in Table 1. Seven hundred lung CT scans of the 269 patients were completed with automatic positioning technology and a relocatable CT system in the Fang Cang Hospital. All scans were completed successfully with the assistance of the automatic positioning technology. Among 700 times camera-based intelligent auxiliary positioning, 642 (91.7%) times got the accurate position lines automatically; only 58 (8.3%) times needed artificial adjustments. Among 700 times positioning box self-adaption, 660 (94.3%) times got the accurate scanning range; 40 (5.7%) times fine adjustments were made. All images (100%) had high quality for reviewing (Table 2). Two CT radiographers and one radiologist worked in this mobile cabin hospital. None of them had any close contact with patients and none of them confirmed COVID-19 during work.

**Table 1:** Demographic characteristics of 269 patients treated in a Fang Cang hospital.

**Basic characteristics**	**All patients (*n* = 269)**
**Sex (%)**	
Female	166 (61.7%)
Male	103 (38.3%)
**Age**	
All population	48.35 ± 12.38
Female group	48.15 ± 12.84
Male group	48.66 ± 11.65
**Age range (%)**	
≤ 20	5 (2%)
21–30	14 (5.2%)
31–40	56 (20.8%)
41–50	71 (26.4%)
51–60	77 (29%)
61–70	44 (16.5%)
>70	2 (0.1%)

**Table 2:** Imaging analysis of 700 scans conducted by re-locatable CT.

	**All images (*n* = 700)**
**Position accuracy**	
Accurate position group	642 (91.7%)
Artificial adjustment group	58 (8.3%)
**Scanning range accuracy**	
Accurate range group	660 (94.3%)
Adjustment group	40 (5.7%)
**Imaging quality (%)**	
High quality	700 (100%)
Poor quality	0

## Discussion

All 269 patients seen at the mobile cabin hospital were all confirmed cases of COVID-19 with mild symptoms. They could move freely and were able to complete the CT examination according to the radiographer's requirements alone. During the entire examination process, the radiographers could monitor the patients through the intelligent camera and communicate with the patient by the voice system. Therefore, it is completely feasible to perform CT examination without contact with patients closely aided by automatic positioning technology.

Complete CT examination usually requires two radiographers. One operates the machine in the controlling room and another positions the patient in the scanning room. By using automatic positioning technology, only one radiographer is needed to perform the CT scan. On the other hand, patients and medical staff go to different areas of the CT room through different channels. Therefore, the design of the relocatable CT and automatic positioning technology system can minimize the close contact between technologists and patients as much as possible, which may reduce or even avoid the occupational exposure of medical staff.

Previous studies demonstrated that CT played a vital role in the screening, diagnosis, and evaluation of treatment response of COVID-19 (16–18). Furthermore, the reported sensitivity of CT is higher than that of RT-PCR in detecting COVID-19 cases (19, 20). Therefore, CT was considered a standard clinical diagnostic tool in China and helped us to screen out suspected cases and evaluate the treatment response of patients. During the outbreak of COVID-2019 in China, the mobile cabin hospital, also called Fang Cang hospital, efficiently helped in controlling the spread of the epidemic by treating enormous numbers of patients with mild symptoms in a short time. In this new treatment mode, the relocatable CT system is a useful equipment for diagnosis due to its convenience of installment. Moreover, since we have reached the post-pandemic era, relocatable CT using automatic positioning technology can be applied for community screening or medical supporting program for remote areas, assisting disease control.

In the routine process of CT examination, medical staff, such as radiographers, need close and frequent contact with different patients. Approximately 3.5% of the confirmed patients with COVID-19 are medical staff (21). Therefore, it is very meaningful to apply automatic positioning technology to relocatable CT in Fang Cang hospitals to minimize the contact between radiographers and confirmed patients. Our study indicated that relocatable CT can effectively protect medical workers from direct exposure of confirmed cases without sacrificing image quality, consisting with other studies (22).

The relocatable CT still exhibited limitation. All patients with COVID-19 in Wuhan Fang Cang Hospital underwent non-contrast pulmonary CT examination. Considering the need for needle docking when injecting contrast agents which requires human participants, the system has not been used in contrast-enhanced examination. For further application in conventional hospitals, the automatic positioning technology may still need an operator to go inside the controlling room for contrast agents. Despite this, the technology can still benefit radiographers from reducing contact with patients, completing accurate examination automatically.

Our study presents some limitations. First, this study applied one radiologist to evaluate the quality of CT images because of the shortage of medical workers in Fang Cang hospitals. Further studies on application of relocatable CT in community screening or medical supporting program for remote areas in the post-pandemic era will be conducted by using several assessors as well as quantitative methods. Also, it is a single-center study and the results of our study shall be improved by cooperating with other centers.

In summary, automatic positioning technology applied to relocatable CT can minimize the close contact between radiographers and patients and effectively improves the protection of medical staff without sacrificing image quality.

## Data Availability Statement

The raw data supporting the conclusions of this article will be made available by the authors, without undue reservation.

## Ethics Statement

The studies involving human participants were reviewed and approved by Medical Ethical Committee of Second Xiangya Hospital (Approved No. 2020002). Written informed consent for participation was not required for this study in accordance with the national legislation and the institutional requirements.

## Author Contributions

ZC, SJ, ZJ, and L-HL designed the research. SJ, ZJ, and KY performed the research and acquired the data. ZC, SJ, and ZJ drafted and wrote the paper. YC, XX, WZ, WS, and JL revised the paper. All authors contributed to the article and approved the submitted version.

## Conflict of Interest

The authors declare that the research was conducted in the absence of any commercial or financial relationships that could be construed as a potential conflict of interest.
